# Dramatic recovery without steroid therapy and withdrawal from insulin therapy in a subject with hyperglycemic and hyperosmolar syndrome and depletion of insulin secretory capacity induced by type 2 autoimmune pancreatitis: A case report

**DOI:** 10.1097/MD.0000000000028609

**Published:** 2022-01-14

**Authors:** Yuichiro Iwamoto, Fuminori Tatsumi, Kenji Kohara, Masashi Shimoda, Shuhei Nakanishi, Tomoatsu Mune, Kohei Kaku, Hideaki Kaneto

**Affiliations:** Department of Diabetes, Endocrinology and Metabolism, Kawasaki Medical School, Japan.

**Keywords:** case report, hyperglycemic and hyperosmolar syndrome, insulin secretory capacity, insulin therapy, type 2 autoimmune pancreatitis

## Abstract

**Introduction:**

Autoimmune pancreatitis (AIP) is characterized by the involvement of autoimmune mechanisms and is classified as type 1, together with infiltration of IgG4-positive cells, and type 2 with poor serological abnormal findings. In clinical practice, AIP is often treated with steroid therapy.

**Patient concerns:**

An 81-year-old Japanese woman had thirst and appetite loss in the previous 5 days; thus, she visited a local doctor. The patient had no abdominal or back pain. She had no history of diabetes mellitus, but at that time blood glucose level and HbA1c were as high as 633 mg/dL and 9.7%, respectively, and she was referred to our institution.

**Diagnosis:**

Based on various clinical findings in this patient, we diagnosed her with hyperglycemic and hyperosmolar syndrome and depletion of insulin secretory capacity induced by type 2 AIP.

**Interventions and outcomes:**

The patient completely recovered without steroid therapy and was withdrawn from insulin therapy.

**Conclusions:**

We should bear in mind the possibility of AIP when the sudden onset of hyperglycemia together with enlargement of the pancreas are observed in subjects without a history of diabetes mellitus.

## 1 Introduction

Autoimmune pancreatitis (AIP) is characterized by the involvement of autoimmune mechanisms and is classified as type 1, together with infiltration of IgG4-positive cells, and type 2 with poor serological abnormal findings. In clinical practice, AIP is often treated with steroid therapy in clinical practice.^[1–5]^ According to a previous retrospective analysis of 36 subjects with AIP, frequent lymphoplasmacytic infiltration and interstitial fibrosis were observed histopathologically.^[5]^ In addition, IgG4-positive cell infiltration was specific to type 1 AIP, whereas changes in the pancreatic head were more frequently observed in type 2 AIP.^[5]^ AIP is a unique type of chronic pancreatitis and has distinctive serological and pathological characteristics. However, once insulin secretory capacity becomes markedly depleted due to glucose toxicity or pancreatitis, it is usually difficult to withdraw from insulin therapy. Here, we present a rare case of hyperglycemic and hyperosmolar syndrome (HHS) and depletion of insulin secretory capacity induced by type 2 AIP in a patient who completely recovered without steroid therapy and withdrew from insulin therapy.

## 2 Case presentation

**Figure 1:**
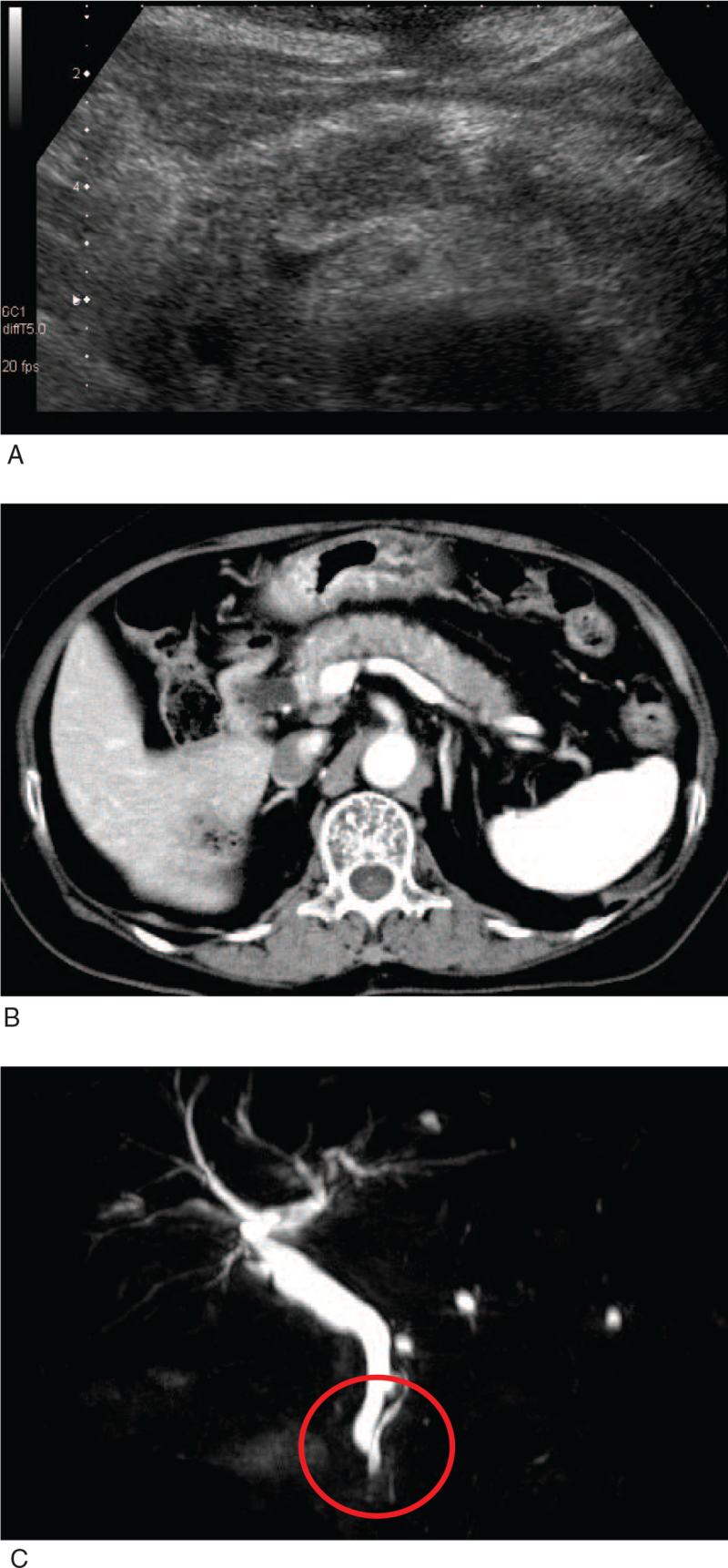
(A) Abdominal ultrasonography. Hypoechoic change of the pancreas was observed, but there was no dilation of pancreatic duct. (B) Abdominal contrast computer tomography. Diffuse swelling of the pancreas accompanied by capsule-like rim was observed. These are characteristic and important findings for the diagnosis of autoimmune pancreatitis. (C) Magnetic resonance cholangiopancreatography. Intrapancreatic common bile duct narrowing and duct type intraductal papillary mucinous neoplasms were suspected. However, there were no tumorous lesions in the pancreas and no dilation in main pancreatic duct.

An 81-year-old Japanese woman experienced thirst and appetite loss in the previous 5 days; thus, she visited a local doctor. The patient had no abdominal or back pain. She had no history of diabetes mellitus (DM), but at that time blood glucose level and HbA1c were as high as 633 mg/dL and 9.7%, respectively. Total ketone bodies and 3-hydoxybutyric acid were also as high as 1582.2 μmol/L and 991.1 μmol/L, respectively. Acidosis was observed as follows: pH, 7.33; HCO_3_^–^, 18.2 mEq/L. She was referred to our institution and hospitalized for treatment of DM and HHS. The patient's height and body weight were 147.4 cm and 51.1 kg, respectively. Her blood pressure and heart rate were 143/85 mmHg and 105/min, respectively. As shown in Table 1, diabetes-associated data on admission were as follows: plasma glucose level, 814 mg/dL and HbA1c, 10.1%. Ketone body levels were increased as follows: total ketone bodies, 1582.1 μmol/L; acetoacetate, 591.1 μmol/L; 3-hydroxybuterate, 991.1 μmol/L. Blood gas analysis data were as follows: pH, 7.33; base excess –6.9 mEq/L. Tests for anti-glutamic acid decarboxylase and anti-IA-2 antibodies yielded negative results. The electrolytes were as follows: sodium, 124 mEq/L; potassium, 5.3 mEq/L; chloride, 89 mEq/L. Renal dysfunction was observed (creatinine, 1.37 mg/dL; blood urea nitrogen level, 40 mg/dL). Liver function was within the normal range. Abdominal ultrasonography revealed a hypoechoic change in the pancreas, but no dilation of the pancreatic duct (Fig. 1A). Abdominal contrast-enhanced computed tomography revealed diffuse swelling of the pancreas, accompanied by a capsule-like rim. These are the characteristic and important findings for the diagnosis of AIP (Fig. 1B). Magnetic resonance cholangiopancreatography revealed intrapancreatic common bile duct narrowing, and duct-type intraductal papillary mucinous neoplasms were suspected. However, there were no tumorous lesions in the pancreas or dilation of the main pancreatic duct (Fig. 1C). In addition, the antinuclear antibody test was positive (640-fold). Since IgG4 was within normal range (IgG4, 12.1 mg/dL) (reference range, 11–121 mg/dL), we excluded the possibility of type 1 AIP. Tissue diagnosis was not performed owing to the potential risk of biopsy. In addition, we did not start steroid therapy because the amylase level did not increase (amylase, 26 U/L) and she had no abdominal or back pain. Therefore, we did not examine the possible responses to steroid therapy. However, based on the above-mentioned findings, we thought that this patient had DM and HHS, both of which were presumably induced by type 2 AIP. It is noted here that we failed to definitively diagnose this subject as type 2 AIP because we did not perform tissue diagnosis and did not examine a possible response to steroid therapy.

**Table 1:** Laboratory data on admission in this subject.

Peripheral blood		Diabetes markers		Electrolytes	
RBC	414 × 10^4^/μL	Plasma glucose	814 mg/dL	Sodium	124 mEq/L
Hemoglobin	13.5 g/dL	HbA1c	10.1%	Potassium	5.3 mEq/L
Hematocrit	39.6%	Glycoalbumin	46.0%	Chloride	89 mEq/L
WBC	9820/μL	Anti-GAD Ab	<5.0 U/mL		
Platelet	38.6 × 10^4^/μL	Anti-IA-2-Ab	<5.0 U/mL	Blood gas	
Blood biochemistry		Total ketone bodies	1582.2 μmol/L	PH	7.330
Total protein	7.6 g/dL	Acetoacetate	591.1 μmol/L	PCO_2_	35.3 mmHg
Albumin	4.9 g/dL	3-Hydroxybuterate	991.1 μmol/L	PO_2_	85.9 mEq/L
Total bilirubin	1.0 mg/dL	Plasma osmolality	315 mOsm/kg	HCO_3_^–^	18.2 mEq/L
AST	11 U/L			Base excess	–6.9 mEq/L
ALT	10 U/L	Lipid markers		Lactate	1.29 mEq/L
LDH	179 U/L	LDL-cholesterol	121 mg/dL		
ALP	254 U/L	HDL-cholesterol	39 mg/dL	Immune markers	
γ-GTP	55 U/L	Triglyceride	194 mg/dL	IgG	803 mg/dL
Creatinine	1.37 mg/dL	LDL/HDL ratio	194 mg/dL	IgG4	12.1 mg/dL
BUN	40 mg/dL	LDL/HDL ratio	3.1	IgA	281 mg/dL
Uric acid	6.1 mg/dL			IgM	51 mg/dL
Amylase	26 U/L			Anti-nuclear Ab	640 fold
CRP	0.47 mg/dL				

γ-GTP = γ-glutamyl transpeptidase, Ab = antibody, ALP = alkaline phosphatase, ALT = alanine aminotransferase, AST = aspartate aminotransferase, BUN = blood urea nitrogen, CPR = C-reactive protein, GAD = glutamic acid decarboxylase, HDL = high density lipoprotein, IA-2 = islet antigen-2, IgG = immunoglobulin, LDH = lactate dehydrogenase, LDL = low density lipoprotein, RBC = red blood cell, WBC = white blood cell.

DM can be complicated by AIP, and endogenous insulin secretion can be improved by intensive insulin therapy. However, most of these cases were of IgG4-related AIP. In this study, we initiated intensive insulin therapy with sufficient intravenous infusion. The maximum insulin doses were 22 units/day and 8 units/day for glulisine and glargine, respectively. Endogenous insulin secretory capacity was evaluated after mitigation of glucose toxicity. It was reduced as follows: CPR index, 0.67; urinary CPR 6.9 μg/day. However, endogenous insulin secretion recovered with improvement in enlargement of the pancreas. About 5 months later, endogenous insulin secretion recovered as follows: FPG 125 mg/dL, CPR index 1.92, CPR 2.4 ng/mL. At that point, the pancreas was not enlarged. Approximately 7 months later, the patient was able to withdraw from insulin therapy. Although endogenous insulin secretory capacity was drastically reduced in this patient, her blood glucose and HbA1c levels were normalized without insulin preparation or any oral antidiabetic agents. Blood glucose and HbA1c levels were approximately 100 mg/dL and 6.0%, respectively.

## 3 Discussion and conclusions

In this report, we present a rare case of HHS with depletion of insulin secretory capacity, both of which were presumably induced by type 2 AIP. The patient completely recovered without steroid therapy and was withdrawn from insulin therapy. To the best of our knowledge, this is the first report showing that type 2 AIP and subsequent HHS can be recovered by intensive insulin therapy without steroid therapy. In addition, we succeeded in withdrawing from insulin therapy by initiating insulin therapy at an early stage.

As described in the case presentation section, ketosis was observed in this patient, but it was not very severe compared to that in patients with typical diabetic ketoacidosis (DK). In addition, the ratio of 3-hydroxybuterate to acetoacetate was 1.68, which was not very high compared to that in subjects with typical DK. In addition, this subject did not consume any food for 5 days and suffered from overt dehydration. Severe mouth dryness was observed in the patient. Therefore, we believe that the increase in ketone body levels in this subject was mainly caused by almost no food intake and severe dehydration. The increase in the anion gap, which was calculated using the measured sodium value, was not very high compared to that in subjects with a typical DK. In addition, we believe that the total ketone body level in this subject was not high enough to lead to an increase in the anion gap. Therefore, we assumed that the increase in anion gap was caused by severe dehydration and moderate ketosis due to starvation. All findings considered, we thought that this subject had HHS, although we failed to completely exclude the possibility of DK. It has been reported that HHS and DK have overlapping pathologies such as ketosis.^[6,7]^ There are also several case reports of HHS together with hyperketonemia.^[8]^

Generally, it is important to perform pancreatic biopsies for the final diagnosis of type 2 AIP.^[9,10]^ Histopathologically, while type 1 AIP is characterized by significant lymphocyte infiltration, IgG4-positive plasma cell infiltration, and storiform fibrosis, type 2 AIP is characterized by the presence of granulocytic epithelial lesions. Type 1 AIP is also called lymphoplasmacytic sclerosing pancreatitis and is recognized as a pancreatic lesion in IgG4-related disease. Type 2 AIP is also called idiopathic duct-centric pancreatitis. In addition, relatively frequent symptoms of AIP include abdominal and/or back pain and weight and/or appetite loss, and relatively frequent complications of AIP are obstructive jaundice and pancreatic endocrine disorders such as DM.

On the other hand, it is well known that pancreas biopsy is accompanied by several potential risks. After careful consideration of the benefits and risks of pancreatic biopsy in this elderly woman, we thought that the benefit would not necessarily outweigh the demerit; thus, we decided not to perform pancreatic biopsy due to its potential risks. Moreover, this patient did not have abdominal or back pain, and the amylase level was within the normal range. Therefore, we avoided starting steroid treatment after careful consideration of the benefits and side effects of this therapy. Although we were missing an anatomopathological report, we were confident of a type 2 AIP diagnosis after assessing the whole clinical and analytical findings of this case. We believe that the above-mentioned time course of medical conditions is an interesting point in this case report: dramatic recovery of AIP without steroid therapy, marked recovery of insulin secretory capacity from insulin secretory depletion, and complete withdrawal from insulin therapy.

Taken together, we should bear in mind the possibility of AIP when the sudden onset of hyperglycemia together with enlargement of the pancreas is observed in subjects without a history of DM. In addition, early intervention with intensive insulin therapy could lead to the preservation of endogenous insulin secretion, even when the insulin secretory capacity is markedly depleted.

## Author contributions

YI, FT, and HK collected the data and wrote the manuscript. KK, MS, SN, TM, and KK contributed to discussion. All the authors have read and approved the manuscript.

**Data curation:** Yuichiro Iwamoto.

**Investigation:** Fuminori Tatsumi, Kenji Kohara, Masashi Shimoda, Shuhei Nakanishi, Tomoatsu Mune, Kohei Kaku, Hideaki Kaneto.

**Writing – original draft:** Yuichiro Iwamoto.

**Writing – review & editing:** Hideaki Kaneto.
